# Caspase-mediated cleavage of raptor participates in the inactivation of mTORC1 during cell death

**DOI:** 10.1038/cddiscovery.2016.24

**Published:** 2016-04-18

**Authors:** R Martin, C Desponds, R O Eren, M Quadroni, M Thome, N Fasel

**Affiliations:** 1 Department of Biochemistry, University of Lausanne, Vaud, Switzerland; 2 Protein Analysis Facility (PAF), Centre Intégratif de Génomique, Quartier UNIL-Sorge, University of Lausanne, Lausanne , Switzerland

## Abstract

The mammalian target of rapamycin complex 1 (mTORC1) is a highly conserved protein complex regulating key pathways in cell growth. Hyperactivation of mTORC1 is implicated in numerous cancers, thus making it a potential broad-spectrum chemotherapeutic target. Here, we characterized how mTORC1 responds to cell death induced by various anticancer drugs such rapamycin, etoposide, cisplatin, curcumin, staurosporine and Fas ligand. All treatments induced cleavage in the mTORC1 component, raptor, resulting in decreased raptor–mTOR interaction and subsequent inhibition of the mTORC1-mediated phosphorylation of downstream substrates (S6K and 4E-BP1). The cleavage was primarily mediated by caspase-6 and occurred at two sites. Mutagenesis at one of these sites, conferred resistance to cell death, indicating that raptor cleavage is important in chemotherapeutic apoptosis.

## Introduction

The mammalian target of rapamycin (mTOR) is an evolutionary conserved protein complex positively regulating anabolic pathways (protein synthesis, energy metabolism, cell survival and cytoskeletal organization) but also repressing catabolic pathways (autophagy and apoptosis). Two different mTOR complexes exist:^1,2^ mTOR complex 1 (mTORC1) and mTOR complex 2 (mTORC2). These two complexes are both composed of the mTOR serine/threonine protein kinase, deptor,^3^ mLST8^4^ and tti1/tel2.^5^ In addition, mTORC1 is composed of specific proteins: the regulatory-associated protein of mTOR (raptor)^6^ and pras40,^7^ whereas mTORC2-specific proteins are the rapamycin-insensitive companion of mTOR (rictor),^8,9^ mSin1^10^ and protor 1 and 2.^11^

Raptor acts as a scaffold protein inside mTORC1, maintaining the dimerization state of the complex^12–14^ and recruiting substrates to the kinase domain of mTOR.^15^ In this context, the initiation of the protein translation machinery is controlled at two different levels by raptor and mTOR. On one hand, raptor binds and recruits the eukaryotic translation initiation factor 4E-binding protein 1 (4E-BP1) to mTORC1, allowing its phosphorylation by mTOR at Thr37/46, which induces the release of 4E-BP1 from the eukaryotic translation initiation factor 4E (elF4E) and gives rise to the activation of cap-dependent mRNA translation.^16,17^ On the other hand, raptor binds to the p70 S6 kinase 1 (p70 S6K1) enabling its phosphorylation by mTOR at Thr389, which induces p70 S6K1 to phosphorylate the S6 ribosomal protein and activate protein synthesis.^18,19^

As a central regulator of cell growth, mTORC1 is frequently hyperactivated in a large proportion of human cancers,^20^ leading to tumorigenesis. This is mainly due to mutations occurring in upstream regulators of mTORC1 (such as RTK, PI-3K, Akt, Erk, PTEN and p53),^1^ giving rise to hyperactive mTORC1, increase in phosphorylation of its downstream targets and thus, enabling abnormal proliferation. In addition, activating mutations have been identified in the *MTOR* gene, leading to hyperactivation of the mTOR pathway.^21^

In this context, the mammalian target of rapamycin has been largely studied as a target for cancer treatments. Inhibitors of mTOR like rapamycin (an allosteric inhibitor) and its analogs (rapalogs) were developed to target this complex. However, the presence of negative feedback loops in the mTOR pathway may have a role in the limitation of treatment efficacy of rapalogs.^22–27^

To counteract this effect, inhibitors of the mTOR kinase activity were developed and unlike rapamycin, a more robust repression of 4E-BP1 phosphorylation was reached with the use of these inhibitors.^24,26^ Recently, new strategies have been developed to target mTORC1 and its upstream regulators at the same time in order to block the oncogenic cascade. Promising results were obtained using dual PI-3K/mTOR inhibitors.^23^

Common chemotherapies against various types of cancer are using etoposide and cisplatin to induce cancer cell apoptosis.^28,29^ Cisplatin is a platinium-based drug creating DNA crosslinking and triggering apoptosis, whereas etoposide is a topoisomerase inhibitor causing DNA strand breaks and promoting apoptosis. These two drugs are also known to affect the mTOR pathway by reducing phosphorylations of 4E-BP1 and S6K.^30–32^

Natural compounds are now emerging as alternative therapies for cancer treatments such as curcumin, the polyphenol compound extracted from rhizome of the plant *Curcuma longa*. This compound has been shown to alter proliferation of cancer cells and also to disrupt the mTORC1 by weakening mTOR–raptor interaction.^33–35^

In an attempt to understand more deeply the molecular mechanisms of anticancer drugs targeting mTORC1, we studied the cell death cascade induced by different drugs on lymphoma cell lines. We report here that rapamycin, cisplatin, etoposide, curcumin, staurosporine (STS) and Fas ligand (FasL) induced caspase-mediated cleavage of raptor and inactivation of the mTORC1, a critical step for apoptosis induction.

## Results

### Raptor is cleaved in various drug-treated lymphoma cell lines

**Figure 1:**
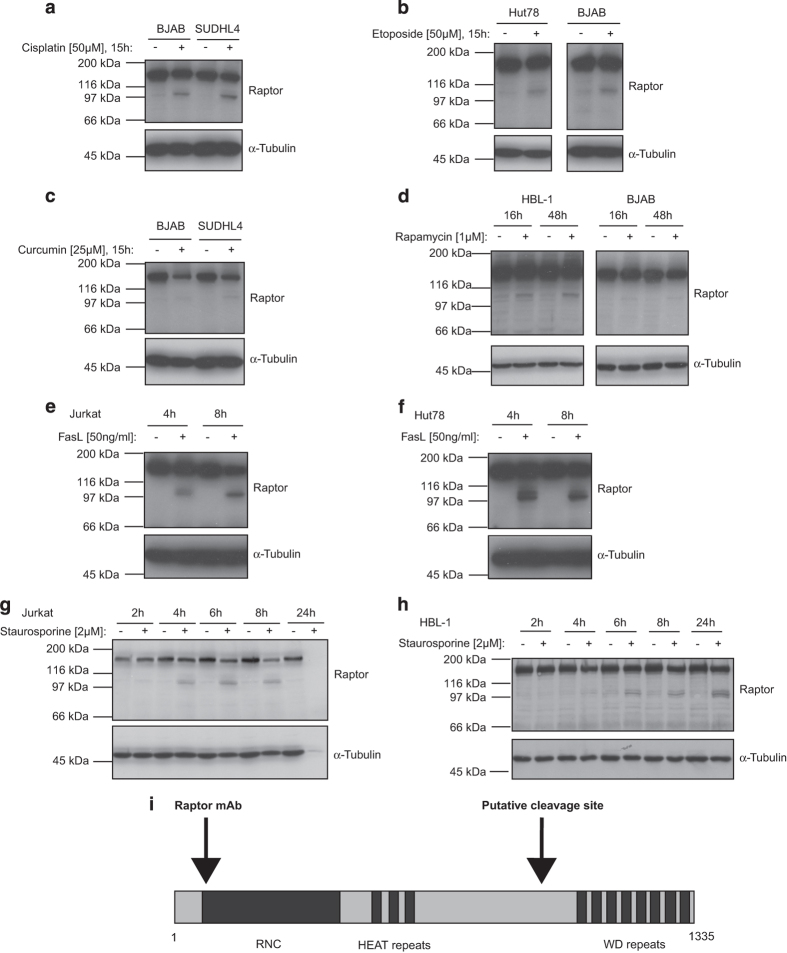
Raptor cleavage upon drug treatment in various T lymphoma and B-cell lines. Lymphoma T (Jurkat, Hut78) or B-cell lines (HBL-1, BJAB, SUDHL4) were incubated with 50 *μ*M of cisplatin for 15 h (**a**), 50 *μ*M of etoposide for 15 h (**b**), 25 *μ*M of curcumin for 15 h (**c**), 1 *μ*M of rapamycin for 16 or 48 h (**d**), 50ng/ml of FasL for 4 or 8 h (**e** and **f**) and with 2 *μ*M of STS for 2, 4, 6, 8 or 24 h (**g** and **h**). Cell lysates were then analyzed on 8% SDS-PAGE. (**i**) Scheme of raptor protein highlighted with the region recognized by the raptor mAb and with the putative cleavage site. RNC, raptor N-term conserved domain; HEAT, Huntingtin, elongation factor 3 (EF3), protein phosphatase 2A (PP2A), and the yeast kinase TOR1; WD, tryptophan-aspartic acid (WD) dipeptide.

To investigate the effect of anticancer drugs on mTORC1, we incubated lymphoma cell lines with cisplatin or etoposide. As shown in Figure 1a, treatment of B lymphoma cell lines (BJAB and SUDHL4) for 15 h with cisplatin induced a cleavage of the full-length raptor protein of 150 kDa into a smaller fragment of approximately 100 kDa. Similar observations were made after 15 h of etoposide treatment in T (Hut78) and B (BJAB) cell lymphomas (Figure 1b).

The regulatory-associated protein of mTOR (raptor) is composed of three evolutionarily conserved domains: a raptor N-terminal (N-term) conserved domain (also called RNC), three HEAT repeats and seven WD repeats, which are protein interaction domains mediating the raptor–mTOR interaction.^14^ This interaction has been previously shown to be weakened after curcumin or high-dose rapamycin treatments.^34,36^ For this reason, we next investigated the effect of curcumin or rapamycin on lymphoma cell lines. After treatment with curcumin or rapamycin, raptor was also cleaved into a 100 kDa fragment in B lymphoma cell lines (Figures 1c and d), supporting the previously reported destabilizing effect of curcumin and rapamycin on mTORC1. As cisplatin, etoposide, curcumin and rapamycin were reported as apoptosis inducers,^33,37–39^ we decided to use two other well-described cell death inducers, STS and FasL, to assess the correlation between cell death and raptor processing.^38,40^ Treatment of T and B lymphoma cell lines with FasL (Figures 1e and f) or STS (Figures 1g and h) both induced a clear processing of raptor into a 100 kDa fragment. Interestingly, we used a rabbit monoclonal antibody (mAb) recognizing the N-term part of raptor, which suggested that, based on the size of cleaved fragment, the observed cleavage occurred within the C-terminal (C-term) part of raptor, probably between the HEAT repeats and the WD repeats (Figure 1i).

Taken together, these data showed that anticancer drug treatments as well as apoptosis inducers gave rise to a cleavage of raptor, an essential scaffold protein of the mTORC1.

### Raptor cleavage is mediated by caspases

**Figure 2:**
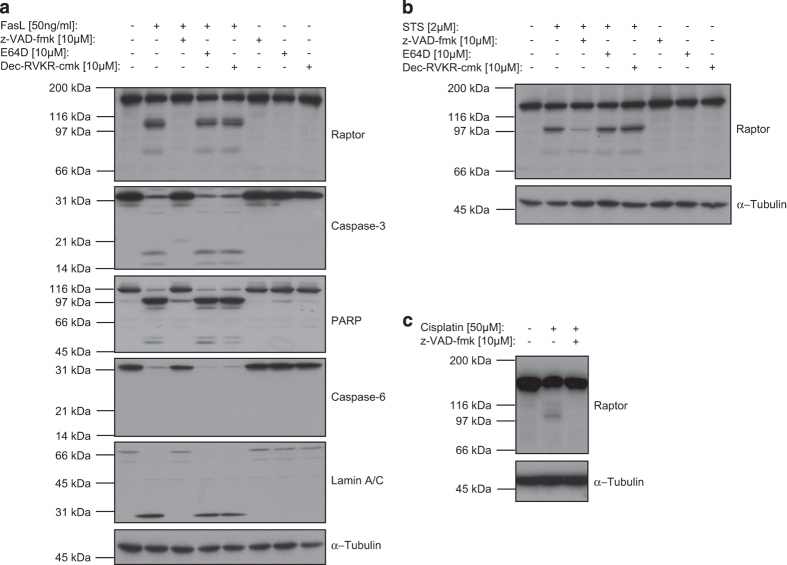
Inhibition of raptor processing by z-VAD-fmk. Jurkat T cells were pre-incubated for 30 min with different inhibitors (z-VAD-fmk, E64D or Dec-RVKR-cmk) and then treated with FasL for 4 h (**a**), STS for 4 h (**b**) or with cisplatin for 16 h (**c**). Cell lysates were then analyzed on 8% or 12% SDS-PAGE.

Different proteases activated during cell death can be responsible for the observed cleavage of raptor. For this reason, we decided to test the following cell permeable protease inhibitors and verify whether they affected raptor cleavage: a general caspase inhibitor (z-VAD-fmk), a cathepsin inhibitor (E64D) and a furin inhibitor (Dec-RVKR-cmk). Jurkat T cells were used as the processing of raptor was easily induced in this cell line. Treatment of Jurkat T cells for 4 h with FasL induced a clear cleavage of raptor into the above-described 100 kDa fragment, and with lower efficiency, into an additional, smaller fragment of 70-80 kDa (Figure 2a). The addition of the caspase inhibitor (z-VAD-fmk) completely abolished the cleavage of raptor and well-known apoptotic events, whereas the cathepsin and furin inhibitors had no effect on the processing. In addition, z-VAD-fmk efficiently inhibited raptor cleavage induced by intrinsic apoptosis inducers: STS or cisplatin (Figures 2b and c).

These results indicated that activation of caspases, by extrinsic or intrinsic apoptosis inducers, mediated raptor processing into molecular species of 100 kDa and 70–80 kDa.

### Active recombinant caspase-1 and caspase-6 can cleave raptor *in vitro*

**Figure 3:**
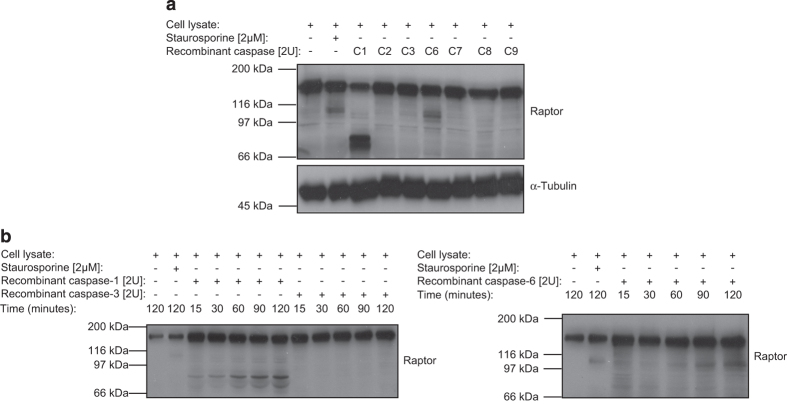
*In vitro* cleavage of raptor by recombinant caspase-1 and -6. (**a**) Jurkat T-cell lysates were incubated with two units of recombinant caspase-1 (C1), caspase-2 (C2), caspase-3 (C3), caspase-6 (C6), caspase-7 (C7), caspase-8 (C8) or caspase-9 (C9) and raptor cleavage was monitored and compared with a STS-treated Jurkat T-cell lysate. (**b**) Time-dependant *in vitro* cleavage of raptor by caspase-1, -3 or -6 in Jurkat T-cell lysates using two units of each recombinant proteins.

To determine more precisely which caspase(s) could be implicated in raptor cleavage, recombinant caspase(s)-1, -2, -3, -6, -7, -8 and -9 were incubated with Jurkat T-cell lysates (Figure 3a). In comparison with cells treated with STS, used a positive control, caspase-6 was the only caspase that induced processing of raptor into the expected 100 kDa band, whereas caspase-1 was able to cleave raptor into two smaller fragments of roughly 70 and 80 kDa. Although recombinant caspase-1 and -6 induced an *in vitro* time-dependant cleavage of raptor in Jurkat T-cell lysates (Figure 3b), activation of the inflammatory caspase-1 in bone marrow-derived macrophages (BMDMø) did not highlighted processing of raptor, suggesting that caspase-1 most likely did not account for physiological raptor cleavage (Supplementary Figure S1).^41^

**Figure 4:**
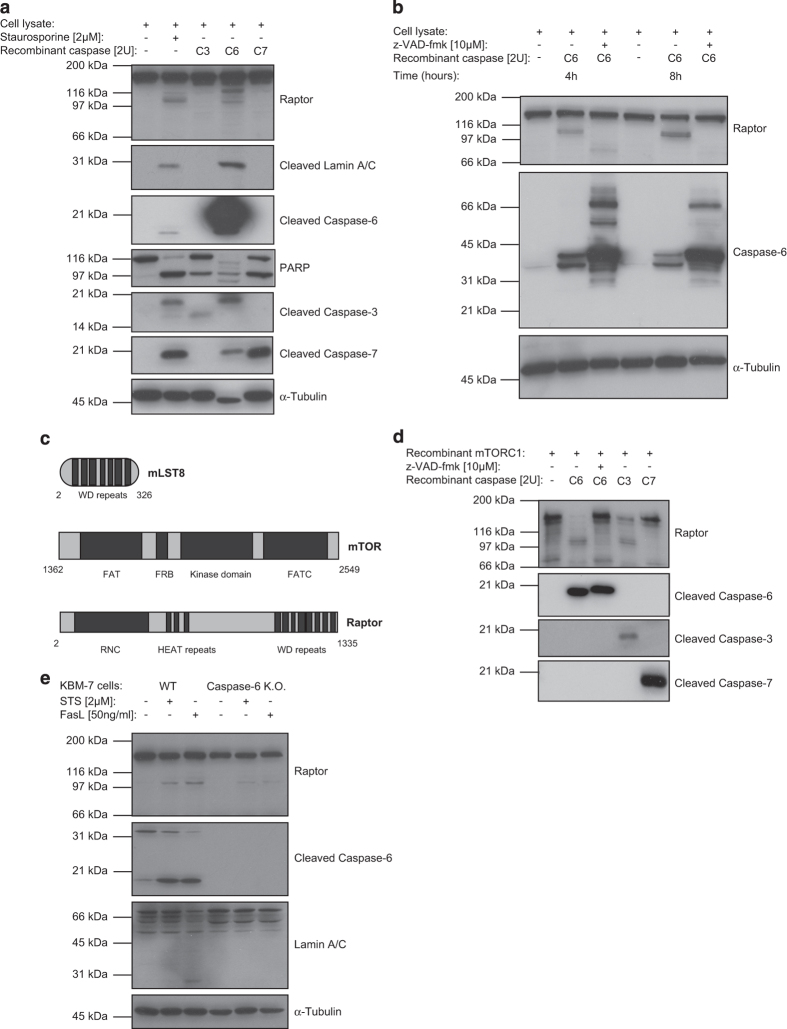
Raptor cleavage by caspase-6 and other caspases. (**a**) Jurkat T-cell lysates were incubated with recombinant caspases-3, -6, -7 for 2 h. Cleavage of raptor and physiological substrates of caspases (PARP and lamin A/C) were monitored and compared with the positive control sample (Jurkat T cells treated with STS for 2 h). (**b**) The effect of z-VAD-fmk on the recombinant caspase-6-mediated cleavage of raptor in Jurkat T-cell lysate. (**c**) Cartoon of recombinant mTORC1 (composed of mLST8, mTOR (1362-end) and recombinant raptor). FAT, focal adhesion targeting; FRB, FKBP12-rapamycin binding; FATC, C-term focal adhesion targeting. (**d**) Cleavage of the full-length recombinant raptor (inside recombinant mTORC1) by recombinant caspase-6, -3 and -7 in the presence (or not) of z-VAD-fmk. (**e**) WT *versus* caspase-6 K.O. KBM-7 cells were treated with STS or FasL for 4 h and then cell lysates were analyzed by SDS-PAGE and western blot.

As recombinant caspase-6 generated similar processing of raptor than treatment with pro-apoptotic drugs, we decided to investigate this processing in more detail. In Figure 4a, the cleavage of the poly (ADP-ribose) polymerase (PARP) by caspase-3 and -7,^42^ and the cleavage of lamin A/C by caspase-6^43,^ ^44^ revealed the specificity of these active recombinant caspases in Jurkat T-cell lysate. As shown before, caspase-6 was the only executioner caspase able to cleave raptor in cell lysates and addition of z-VAD-fmk abolished processing of raptor, confirming that the cleavage was depending on the catalytic activity of recombinant caspase-6 (Figures 4a and b).

To clarify whether the processing of raptor obtained with recombinant caspases occurred directly or in two steps, as a consequence of activation of another protease in the lysates by these recombinant caspases, we used a recombinant mTORC1 composed of essential proteins required for mTOR activity (Figure 4c). The cleavage of raptor was observed in the presence of caspase-6 and also with caspase-3, yielding a fragment with the same molecular mass (100 kDa) than observed after apoptosis induction. This processing was completely abolished after addition of z-VAD-fmk. Cleavage of full-length raptor (150 kDa) was less strong with caspase-3 when compared with caspase-6, and caspase-7 was not able to cleave recombinant raptor inside the mTORC1 (Figure 4d).

Taken together, these results showed that recombinant caspase-6 efficiently cleaved raptor in Jurkat T-cell lysates as well as recombinant raptor inside mTORC1. Whereas raptor could not be cleaved in cell lysates by other recombinant caspases such as caspase-3 and -7, recombinant caspase-3 was able to cleave recombinant raptor inside mTORC1, suggesting that different caspases could be implicated in raptor processing.

### Caspase-6 has a role in raptor processing *in vivo* under apoptotic conditions

To confirm *in vivo* caspase-6-mediated processing of raptor, we used a caspase-6 knockout (KO) chronic myelogenous leukemia cell line (KBM-7) generated by gene trapping (Figure 4e). As expected, the cleavage of caspase-6 and lamin A/C were only observed in WT cells after STS or FasL treatments and not in caspase-6 KO cells. Raptor processing was clearly reduced (but not completely abolished) in caspase-6 KO compared with WT KBM-7 cells. This suggests that caspase-6 has a major role in raptor processing *in vivo*, but other caspases most likely contribute to this process.

### Raptor is cleaved at the C-terminus part after DDADD residues

**Figure 5:**
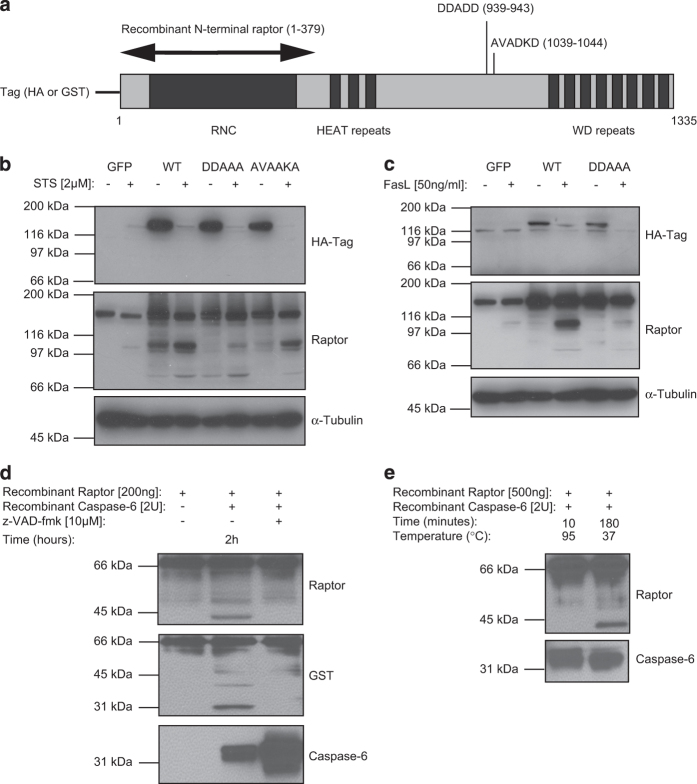
Cleavage of raptor at the C-term DDADD (amino acids 939–943) residue and at the N-term DEADLTD (amino acids 17–23) residue. (**a**) Cartoon of raptor and its putative C-term caspase-mediated cleavage residues (UniProtKB: Q8N122). The recombinant N-term raptor protein (amino acids 1–379) is represented by the arrow. Different N-term tags (HA or GST) were used according to the raptor constructs. (**b**) Jurkat T cells were transfected with GFP, WT, DDAAA or AVAAKA raptor and treated with STS for 2 h. (**c**) Jurkat T cells were transfected with GFP, WT or DDAAA raptor and treated with FasL for 2 h. (**d**) Recombinant N-term raptor was incubated with recombinant caspase-6 for 2 h in the presence (or in the absence) of z-VAD-fmk. (**e**) Recombinant raptor was incubated with recombinant caspase-6 and directly inactivated at 95 °C for 10 min or incubated for 3 h at 37 °C. The samples were analyzed by tandem MS.

It is known that caspases are exclusively recognizing the negatively charged aspartic acid (or aspartate (Asp, D)) residues in the P1 position of the cleavage site. Two potential cleavage sites, corresponding to the size of the cleaved fragment, were found within the C-term part between the HEAT repeats and the WD repeats: DDADD (UniProtKB: Q8N122, amino acids 939–943) and AVADKD (amino acids 1039–1044) (Figure 5a). To understand if these residues were involved in the caspase-mediated cleavage of raptor, Jurkat T cells were transiently transfected by electroporation with raptor WT or mutated constructs (DDAAA or AVAAKA) (Figures 5b and c). Similar expression levels were achieved for the WT, DDAAA and AVAAKA raptor constructs detected via an HA tag at the N-terminus or via an anti-raptor antibody, which detected both the endogenous raptor polypeptide and the overexpressed raptor constructs. The processing of raptor was sometimes evident already in non-treated cells for the WT construct, possibly as a consequence of the stress induced by the transfection, but it was further increased by STS treatment (Figure 5b). On the other hand, the cleavage of the DDAAA construct was clearly reduced compared with the WT or the AVAAKA raptor constructs, in both treated and untreated cells. A similar decrease in raptor cleavage was observed with the DDAAA *versus* the WT raptor constructs after FasL treatment (Figure 5c). Interestingly, after overexpression of raptor WT or mutated constructs in Jurkat T cells, an additional cleavage fragment appeared at approximately 70–80 kDa after cell death induction (Figures 5b and c). This faster migrating cleaved band could correspond to a lower efficiency caspase-mediated cleavage site.

Taken together, these experiments highlighted the importance of the DDADD, amino-acid 939–943 residues (located within the raptor C-term region, between the HEAT repeats and the WD repeats), in the caspase-mediated processing of raptor.

### Raptor is cleaved at the N-terminus part after DEADLTD residues

As shown in Figures 5b and c, the HA tag signal disappeared after STS or FasL treatment, possibly due to activation of caspases that could cleave inside the HA tag sequence^45^ and/or because of the presence of a raptor N-term cleavage site. To determine the presence of a potential N-term raptor cleavage site, we used a recombinant glutathione-*S*-transferase (GST)-raptor protein. This recombinant protein has a molecular mass of 70 kDa and contains the N-term sequence of raptor (amino acid 1–379) fused to the N-terminus with GST (Figure 5a). Incubation of the N-term recombinant raptor with recombinant caspase-6 generated a cleavage of raptor from the 70 kDa into a molecular species of 40 kDa, indicating the presence of a caspase-6-mediated cleavage site at the beginning of the raptor sequence, after the GST tag (Figure 5d).

Next, two samples were prepared to identify the raptor N-term cleavage site(s) by mass spectrometry (MS) (Figure 5e). A tryptic peptide with sequence LTDWNLPLAFMK was clearly detected, which resulted from cleavage after an aspartate residue in the sequence DEAD (amino-acid 20) (Supplementary Figure S2). In addition, another potential caspase-6-specific cleavage site (DEADLTD, amino-acid 23) was found next to the DEAD site. This potential cleavage site seemed less prone to be cleaved by caspase-6, as it appeared in much lower amounts as judged by spectral counts (Supplementary Figure S2). Moreover, recombinant caspase-3 or -7 were also able to process the N-term recombinant raptor into a 41 kDa fragment, similary to recombinant caspase-6 (Supplementary Figure S3).

Taken together, these data indicated that raptor could be cleaved *in vitro* by caspase-6 (and by caspase-3 and -7) after two N-term aspartate residues localized next to each other as present in the DEADLTD sequence.

### Raptor processing correlates with mTORC1 inhibition and cell death

**Figure 6:**
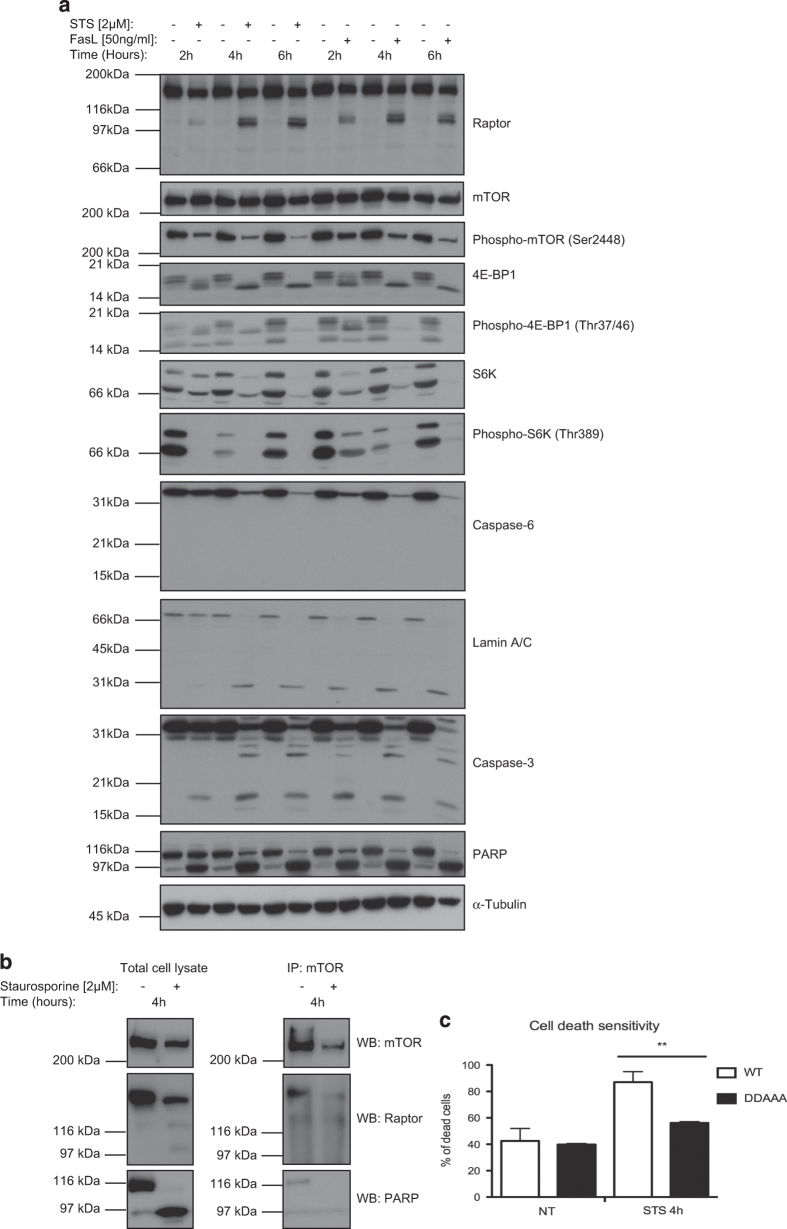
Inhibition of mTORC1 and cell death sensitivity caused by raptor processing and decrease of mTOR-raptor interaction. (**a**) Jurkat T cells were treated with STS or FasL for 2, 4 or 6 h. Raptor processing, phosphorylation of mTORC1 substrates (S6K, 4E-BP1), caspase-3 and -6 activations were analyzed. (**b**) Untreated or STS-treated Jurkat T-cell lysates were IP with an antibody raised against mTOR. Raptor–mTOR interaction, PARP and raptor cleavage were analyzed. (**c**) Jurkat T cells were transfected with WT or DDAAA raptor constructs and treated with STS for 4 h. A live/dead staining was used to quantify the percentage of cell death by flow cytometry. The unpaired Student’s *t*-test (Prism software) was used to calculate the significative difference. **P=0.0025. Data are represented as mean±S.E.M.; *n*=3.

As raptor has an important role as a scaffold protein within mTORC1, the processing of raptor could have a negative effect on mTORC1 structure and activity. To assess whether the processing of raptor led to the inhibition of mTORC1 signaling, the downstream targets of mTORC1 were analyzed for phosphorylation, as well as the phosphorylation of mTOR at Ser2448 by an upstream activator, Akt.^46,47^ Incubation of Jurkat T cells with STS or FasL for 2, 4 and 6 h led to progressive cleavage of raptor, whereas phosphorylation levels of mTOR, 4E-BP1 and S6K were all decreasing after incubation with STS or FasL (Figure 6a). As STS is a protein kinase inhibitor, a decrease in multiple phosphorylation events was expected. However, FasL has no direct effect on protein kinase activity and nevertheless, the same decrease of phosphorylation of mTOR substrates was observed, suggesting that the effect on their phosphorylation was linked to the processing of raptor. Thus, activation of caspase-3 and -6, as well as cleavage of their respective substrates (PARP, lamin A/C), correlated perfectly with cleavage of raptor and with loss of phosphorylation of 4E-BP1 and S6K.

Although raptor is essential for binding and recruiting mTOR substrates,^15^ several studies showed that treatment with curcumin or with high concentrations of rapamycin induced the weakening of the raptor–mTOR interaction,^34,36^ which could affect the mTORC1 activity. Figure 6b showed by immunoprecipitation (IP) that mTOR was bound to raptor in the untreated sample, but the interaction was strongly reduced in the STS-treated sample and no processed band was observed at 100 kDa after IP, suggesting that raptor cleavage weakens the mTOR–raptor interaction and that the cleaved raptor is probably released from the complex.

As the mutation of the C-term raptor cleavage site abolished raptor processing after apoptosis induction, and the interaction between raptor and mTOR was altered when raptor was cleaved, we hypothesized that the absence of raptor processing could lead to a difference in cellular survival under stress conditions. For this reason, we investigated the effect of overexpressing a cleavage-resistant mutant of raptor (DDAAA) *versus* WT raptor on apoptosis induction (Figure 6c). Apoptosis induction with STS had a strong effect on cells transfected with the raptor WT construct (almost 90% of dead cells), whereas cells transfected with cleavage-resistant raptor (DDAAA) showed a significative reduction in STS-induced cell death (nearly 60%). Thus, raptor cleavage had an important role in the apoptotic cascade, as the abolition of its processing induced resistance against cell death.

Taken together, these results suggested that raptor processing correlated with its dissociation from mTORC1, loss of phosphorylation of mTOR substrates and thus, inactivation of the mTORC1 during apoptosis.

## Discussion

In this study, we investigated the molecular mechanisms involved in the targeting of mTORC1 by anticancer drugs, as this complex is frequently hyperactivated in human cancers. Treatment of different lymphoma T- and B-cell lines with anticancer drugs like cisplatin, etoposide, rapamycin, curcumin and apoptosis inducers like STS and FasL led to the processing of the 150 kDa raptor protein into a smaller fragment of 100 kDa. Interestingly, rapamycin is known as an allosteric inhibitor of mTORC1, which, at low concentrations, binds to the intracellular protein FKBP12 to form a gain-of-function complex, inducing inhibition of mTORC1. Repression of mTORC1 by low dose of rapamycin will give rise to induction of autophagy, as mTORC1 normally blocks initiation of autophagy through Unc-51 like autophagy activating kinase 1 (ULK1) inhibition. Contrariwise, high doses (defined as 20 *μ*M) of rapamycin were shown to induce apoptosis (cleavage of PARP) as well as mTOR-raptor dissociation and loss of phosphorylation of 4E-BP1.^36,39^ The precise mechanism of action of rapamycin (at high dose) has not been described but our results can explain the missing gap, as we showed that rapamycin (as well as other drugs) led to caspase activation (Supplementary Figures S4 and S5), which cleaved raptor, dislocated and therefore inhibited the mTORC1. In our hands, both high and low concentrations of rapamycin gave rise to processing of raptor and activation of caspases in different cell lines and with varying kinetics (Figure 1d and Supplementary Figure S5). In addition, drugs triggering either the intrinsic (cisplatin, etoposide, STS) or the extrinsic (FasL) apoptosis cascades induced raptor processing, which indicated that different cell death stimuli converged to raptor cleavage.

Here we identified caspase-6 as a key protease involved in the cleavage of raptor at the C-term and at the N-term, but other caspases could have a redundant role, as some processing of raptor was still present in caspase-6 KO cells. This is coherent considering the importance of mTORC1 as a major regulator of cellular anabolic processes. Therefore, different caspases most likely act together to process raptor, inhibit the mTORC1 and thus block cell growth.

The identification of caspase-mediated raptor cleavage sites revealed that the C-term cleavage site was located between the HEAT repeats and the WD repeats, whereas the N-term cleavage site was situated at the beginning of raptor N-term conserved (RNC) domain. The 3-dimensional (3D) structure of the mTORC1 has been resolved using cryo-electron microscopy and revealed the dimeric aspect of the complex.^12,13^ In fact, raptor is situated at the interface between two mTOR molecules and the overall structure of the complex is forming a circle-like shape. Based on this 3D structure, the C-terminus of raptor is situated in between two mTOR proteins, but the N-terminus of raptor is free and located at the outside of mTORC1, probably to access more easily mTOR substrates such as 4E-BP1 and S6K. We would expect that processing after the DDADD C-term cleavage site destabilizes the complex, as this site is situated at the point of contact with two mTOR proteins. The N-term DEADLTD raptor cleavage site could affect the binding of 4E-BP1 and S6K to raptor and thus, reduce their phosphorylation. Furthermore, a previous study highlighted that mutagenesis in the RNC or in the WD repeats of raptor gave rise to a loss of the mTOR–raptor interaction.^14^ Thus, raptor cleavage, which removes a part of the RNC domain and the WD repeats, could abolish this interaction as well. This hypothesis was clearly supported by our experiments, which showed a correlation between raptor cleavage, decrease of raptor-mTOR binding and loss of phosphorylation of mTOR, S6K and 4E-BP1 (Figures 6a and b). Moreover, cleavage of raptor in its C-term region could induce a loss of phosphorylation via the release of the cleaved raptor fragment from mTORC1. In this scenario, the processed portion of raptor (unbound and outside mTORC1) could compete with the remaining unprocessed raptor (bound and inside mTORC1) for mTOR substrates and thus, decrease the amount of phosphorylated S6K and 4E-BP1.

**Figure 7:**
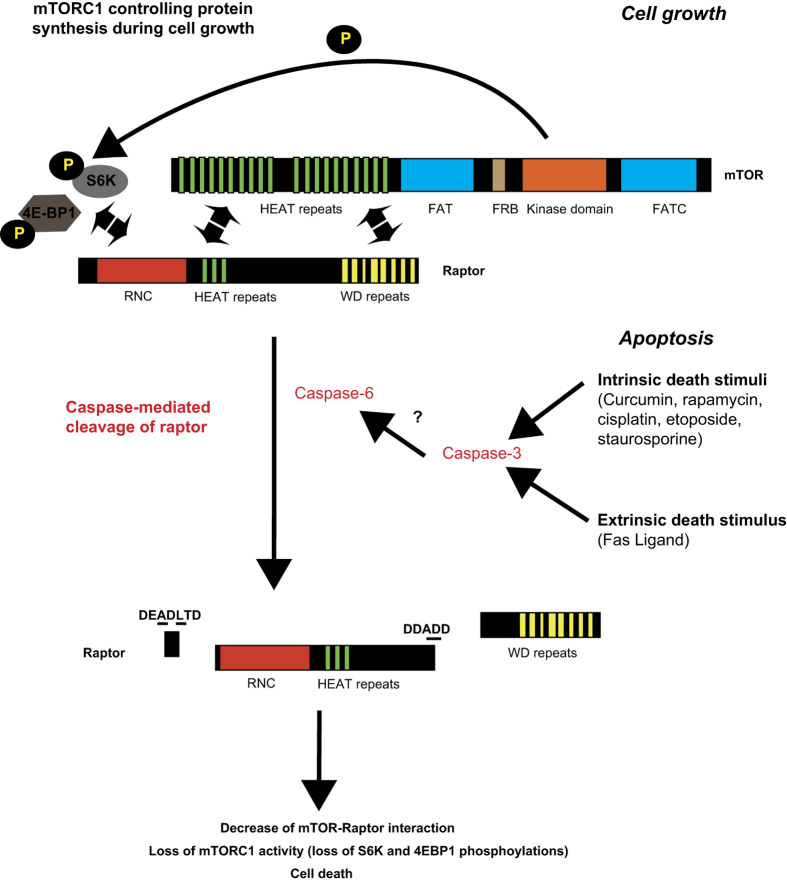
Model representing mTORC1 modulation from cell growth to apoptosis.

Interestingly, the cleavage of raptor had a strong effect at the cellular level, as the transfection of the cleavage-resistant raptor mutant (DDAAA) conferred resistance to cell death compared with transfection with WT raptor. This indicated that the processing of raptor was required during the apoptosis cascade to properly deactivate the mTORC1 function and stop cell growth. Collectively, our findings support a model (Figure 7) in which either intrinsic or extrinsic apoptosis pathways lead to activation of executioner caspases (caspase-3, -6 and -7), which cleave raptor at two specific cleavage sites (at least). These cleavage events give rise to a decrease in the raptor–mTOR interaction and the release of the cleaved raptor from the mTORC1. The mTORC1 is dislocated and inactivated (loss of mTOR substrates phosphorylation), which is crucial to execute the cell death cascade.

An important consequence of our findings is that the processing of raptor can now be used as a new marker of apoptosis and as a complementary indicator of mTORC1 inhibition. As phosphorylations are sometimes difficult to interpret, the cleavage of raptor is a novel precise indicator of mTORC1 inactivation, caspase activation and efficient triggering of the cell death cascade. Novel strategies are required to target the mTORC1, circumvent drug-resistance and stop cancer progression. Our study may also have direct applications for drug screening, as the cleavage of raptor can be used as a selecting marker for the identification of mTORC1-targeting drugs.

## Materials and Methods

### Antibodies, reagents and drugs

Anti-raptor (cat. no. 2280), anti-mTOR (cat. no. 2983), anti-phospho-mTOR Ser2448 (cat. no. 5536), anti-4E-BP1 (cat. no. 9452), anti-phospho-4E-BP1 Thr37/46 (cat. no. 2855), anti-p70 S6K (cat. no. 9202), anti-phospho-S6K Thr389 (cat. no. 9234), anti-cleaved-caspase-7 (cat. no. 9491), anti-cleaved-caspase-6 (cat. no. 9761), anti-caspase-6 (cat. no. 9762), anti-caspase-3 (cat. no. 9662), anti-PARP (cat. no. 9542), anti-lamin A/C (cat. no. 2032) and anti-IL-1β (cat. no. 12242) antibodies were purchased from Cell Signaling Technologies (Danvers, MA, USA). Anti-caspase-1 (cat. no. SC514) antibody was obtained from Santa Cruz Biotechnology (Dallas, TX, USA). Anti-α-tubulin (cat. no. T6074) and anti-HA Tag (cat. no. H3663) were ordered from Sigma (St. Louis, MO, USA).

Cisplatin (cat. no. ALX-400-040), etoposide (cat. no. BML-GR307-0100), rapamycin (cat. no. BML-A275-0005), STS (cat. no. ALX-380-014-M001) and Dec-RVKR-cmk (cat. no. ALX-260-022) were all ordered from Enzo Life Sciences (Farmingdale, NY, USA). Curcumin (cat. no. C1386) and E64D (cat. no. E3132) were purchased from Sigma. The irreversible, cell permeable broad-spectrum caspase inhibitor (z-VAD-fmk, cat. no. N-1510-0005) was obtained from Bachem (Bubendorf, Switzerland). Active recombinant caspases-1, -2, -3, -6, -7, -8, -9 (cat. no. BV-K232-2) were ordered from MBL (Woburn, MA, USA). Recombinant human mTOR/Raptor/mLST8 (cat. no. SRP0364) was purchased from Sigma. Recombinant N-terminus Raptor (cat. no. ab112419) was purchased from Abcam (Cambridge, UK). The recombinant FasL (Fc:FasL) was kindly provided by Professor Pascal Schneider.^40^ The sequencing grade modified trypsin (cat. no. V5111) was obtained from Promega (Dübendorf, Switzerland).

### Cells and cell culture conditions

KBM-7 WT and caspase-6 deficient cells (cat. no. P01285E07) were purchased from Horizon-Genomics (Cambridge, UK). T and B lymphoma cell lines were cultured in RPMI-1640 (Gibco, Carlsbad, CA, USA) supplemented with 10% FCS, 1% HEPES and 1% penicillin (10 000 IU/ml) and streptomycin (10 000 *μ*g/ml). KBM-7 cell lines were maintained in IMDM (Gibco) supplemented with 10% FCS, 1% HEPES and 1% penicillin (10 000 IU/ml) and streptomycin (10 000 *μ*g/ml). Cells were cultured at 37 °C with 5% CO_2_ and splited every 2 days.

### Drug treatments and western blot

Cells were seeded in 12-well plates at 10^6^ cells/ml in 1 ml in culture medium and drugs were added for various period of time. Cells were recovered, spun down for 5 min at 500×*g* and pellets were resuspended in 1x RIPA buffer (Bio Basic Inc., Markham, Canada)/cat. no. RB4478) containing protease/phosphatase inhibitors (Cell Signaling Technologies/cat. no. 5872S) and kept on ice for lysis for 20 min. Cleared cell lysates were obtained after 5-min centrifugation at 14 000×*g*. Cell lysates were dosed for protein concentration using a BCA protein assay kit (Thermo Scientific, Zug, Switzerland)/cat. no. 23225). Thirty microgram of protein was loaded on 8% or 12% SDS-PAGE. Standard western blot procedure was followed.

### *In vitro* caspase reaction

Jurkat T cells (5×10^5^ cells per condition) were lysed in 10 *μ*l of 1x RIPA buffer without protease/phosphatase inhibitors. Cell lysate was incubated in 30 *μ*l of 1x caspase reaction buffer (50 mM HEPES, pH 7.2, 50 mM NaCl, 0.1% CHAPS, 10 mM EDTA, 5% glycerol and 10 mM DTT) with two units of various recombinant caspases. The reaction was performed at 37 °C for 2 to 4 h. Reactions were stopped by the addition of sample buffer and boiling of the sample for 5 min at 95 °C. For the *in vitro* caspase reactions performed on recombinant mTOR/Raptor/mLST8 or on recombinant N-terminus Raptor, 1–2 *μ*g of recombinant protein were added directly into the caspase reaction buffer in the presence of recombinant caspases.

### Caspase-1 activation in BMDMø

BMDMø were generated from C57BL/6 mice using standard protocol.^41^ BMDMø were plated in 48-well plates at 2.5×10^5^ cells per well. The priming step was performed with ultrapure LPS (Labforce, Muttenz, Switzerland) (100 ng/ml) for 3 h. Following priming, the inflammasome was activated with 5 *μ*M Nigericin (Sigma) or 5 mM ATP for 1 h. Supernatants were collected and proteins were precipitated using methanol–chloroform protocol. Cell lysates were collected using 1x RIPA buffer.

### MS analysis

Recombinant N-terminus raptor (500 ng) was incubated with two units (U) of recombinant caspase-6 and boiled for 10 min at 95 °C or incubated at 37 °C for 3 h. Samples were run on a 10% SDS-PAGE and stained overnight with colloidal Coomassie blue and then destained. Interesting bands were cut and partially digested with trypsin (1 h 30-min incubation) into peptides, which were then analyzed by nano-HPLC and high-resolution tandem MS (nanoLC-MS/MS). The database searching software MASCOT (Boston, MA, USA) was used to match the generated spectra to known protein sequences with a mass tolerance of 10 p.p.m. for the precursor peptide mass and 0.5 Da for tandem MS fragments. Cleavage specificity for peptide matching was semi-tryptic (only one cut C-term to Lys and Arg was required).

### Raptor vectors

HA WT raptor plasmid was a gift from David Sabatini (Addgene plasmid # 8513, Cambridge, MA, USA).^14^ Raptor mutants (DDAAA/AVAAKA) were generated by Genscript (Piscataway, NJ, USA).

### Transfections

Jurkat T cells were transfected using Amaxa Cell line Nucleofector Kit V (Lonza, Visp, Switzerland/cat. no, VCA-1003) following the manufacturer’s protocol. After overnight incubation at 37 °C with 5% CO_2_, fresh RPMI was added on the cells for another 24-h incubation. Fourty-eight hours post transfections, cell death was induced either with FasL (50 ng/ml) or STS (2 *μ*M) for 2–4 h.

### Immunoprecipitation

Six million Jurkat T cells were treated with 2 *μ*M of STS for 6 h or left untreated. Cells were washed once in PBS and resuspended in a lysis buffer containing 20 mM Tris (pH 7.5), 150 mM NaCl, 1 mM EDTA, 0.3% CHAPS and 1/100 protease/phosphatease inhibitor cocktail (Cell Signaling Technologies, cat. no. 5872S). Cells were left 20 min on ice and centrifuged at 14 000×*g* for 5 min to obtain cleared cell lysates. Cell lysates were incubated with gentle rocking with anti-mTOR antibody (Cell Signaling Technologies, cat. no. 2983, dilution 1/50) for an overnight incubation at 4 °C. The next day, protein A/G PLUS-agarose beads (Santa Cruz Biotechnology, cat. no. sc-2003) was added to the cell lysate/antibody mix for an additional 3-h incubation with gentle rocking at 4 °C. Then beads were washed with lysis buffer five times, SDS sample buffer was added, samples were heated for 5 min at 95 °C and loaded on a 6% SDS-PAGE for wertern blot analysis.

### Cell death sensitivity test

Fourty-eight hours post transfection, cells were treated with STS (2 *μ*M) for 4 h or left untreated. Then cells were stained using the LIVE/DEAD fixable far red dead cell stain kit (cat. no. L10120, Invitrogen, Waltham, MA, USA) following the manufacturer’s protocol. Cell death was quantified by flow cytometry (Accuri C6 flow cytometer, Ann Arbor, MI, USA). Statistical significance of differences was calculated using unpaired Student’s *t*-test with the Prism software (Irvine, CA, USA).
