# Supplementary material for: Caspase-mediated cleavage of raptor participates in the inactivation of mTORC1 during cell death
Source: Cell Death Discov. 2016 Apr 18;2:16024–. doi: 10.1038/cddiscovery.2016.24 (PMC4979510; doi:10.1038/cddiscovery.2016.24)
Supplement: Supplementary Figure 1 [file cddiscovery201624-s1.pdf]

**Figure S1**

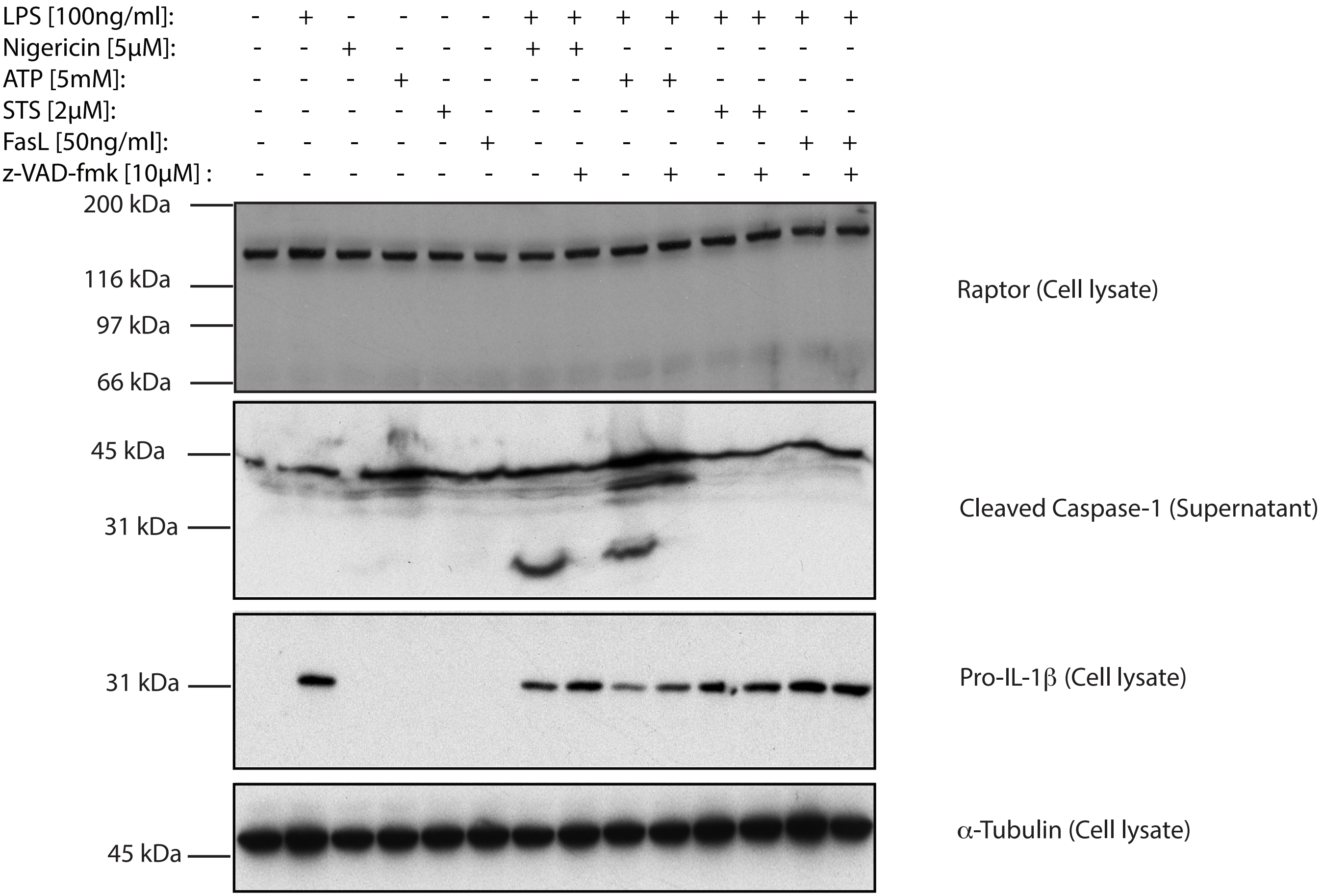

LPS [100ng/ml]: - + - - - - + + + + + + + +
Nigericin [5µM]: - - + - - - + + - - - - - -
ATP [5mM]: - - - + - - - - + + - - - -
STS [2µM]: - - - - + - - - - - + + - -
FasL [50ng/ml]: - - - - - + - - - - - - + +
z-VAD-fmk [10µM] : - - - - - - - + - + - + - +
200 kDa
116 kDa
97 kDa
66 kDa
Raptor (Cell lysate)
45 kDa
31 kDa
Cleaved Caspase-1 (Supernatant)
31 kDa
Pro-IL-1β (Cell lysate)
45 kDa
α-Tubulin (Cell lysate)
