# Supplementary material for: Caspase-mediated cleavage of raptor participates in the inactivation of mTORC1 during cell death
Source: Cell Death Discov. 2016 Apr 18;2:16024–. doi: 10.1038/cddiscovery.2016.24 (PMC4979510; doi:10.1038/cddiscovery.2016.24)

Figure S2

A

Search parameters
Enzyme: semiTrypsin: cuts C-term side of KR unless next residue is P.
Cleavage is semi-specific. (Peptide can be non-specific at one terminus only.)
Fixed modifications: Carbamidomethyl (C)
Variable modifications: Deamidated (NQ), Oxidation (M)
Protein sequence coverage: 69%

Matched peptides shown in bold red.

1	MESEMLQSPL	LGLGEED**EAD**	**LTDWNLPLAF**	**MK**KRHCEKIE	GSK**SLAQSWR**
51	**MK**DRMKTVSV	ALVLCLNVGV	DPPDVVKTTP	CAR**LECWIDP**	**LSMGPQKALE**
101	**TIGANLQKQY**	**ENWQPR**AR**YK**	**QSLDPTVDEV**	**KK**LCTSLRRN	AKEER**VLFHY**
151	**NGHGVPRPTV**	**NGEVWVFNK**N	YTQYIPLSIY	DLQTWM**GSPS**	**IFVYDCSNAG**
201	**LIVKSFKQFA**	**LQREQELEVA**	**AINPNHPLAQ**	**MPLPPSMKNC**	**IQLAACEATE**
251	**LLPMIPDLPA**	**DLFTSCLTTP**	**IK**IALRWFCM	QK**CVSLVPGV**	**TLDLIEK**IPG
301	RLNDR**RTPLG**	**ELNWIFTAIT**	**DTIAWNVLPR**	DLFQK**LFRQD**	**LLVASLFRNF**
351	**LLAER**IMR**SY**	**NCTPVSSPRL**	**PPTYMHAMW**		

B

Query	Start	Peptide	PSM (incl oxidised forms)	Best Mascot Score
42164	18-32	D.EADLTDWNLPLAFMK.K *	1	34
**26882**	**21-32**	**D.LTDWNLPLAFMK.K**	**24**	**63**
10186	24-32	D.WNLPLAFMK.K	2	28
3455	125-132	D.PTVDEVKK.L	2	53
5195	162-169	N.GEVWVFNK.N	2	36
46011	187-204	M.GSPSIFVYDCSNAGLIVK.S	1	56
35187	191-204	S.IFVYDCSNAGLIVK.S	2	43
21140	193-204	F.VYDCSNAGLIVK.S	1	45
49272	219-238	E.VAAINPNHPLAQMPLPPSMK.N	2	32

C

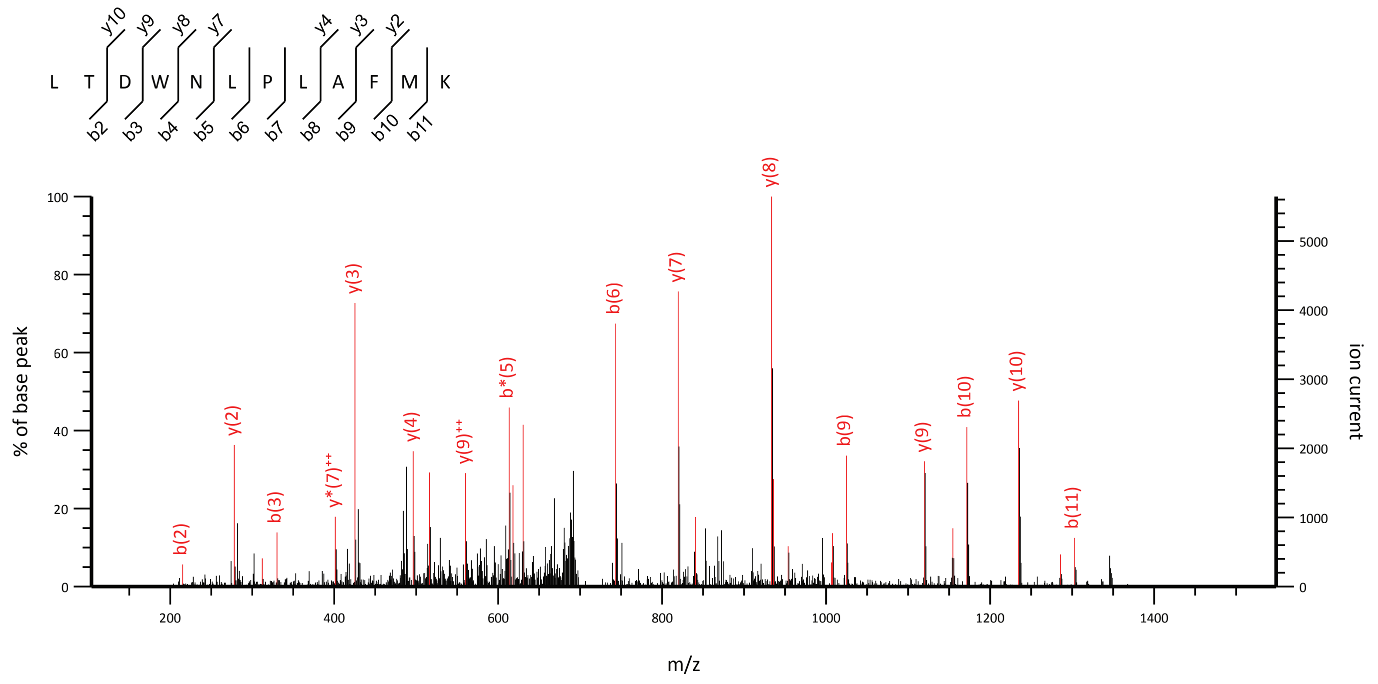

Supplement: Supplementary Figure 2 [file cddiscovery201624-s2.pdf]
