# Supplementary material for: Caspase-mediated cleavage of raptor participates in the inactivation of mTORC1 during cell death
Source: Cell Death Discov. 2016 Apr 18;2:16024–. doi: 10.1038/cddiscovery.2016.24 (PMC4979510; doi:10.1038/cddiscovery.2016.24)
Supplement: Supplementary Figure 3 [file cddiscovery201624-s3.pdf]

Figure S3

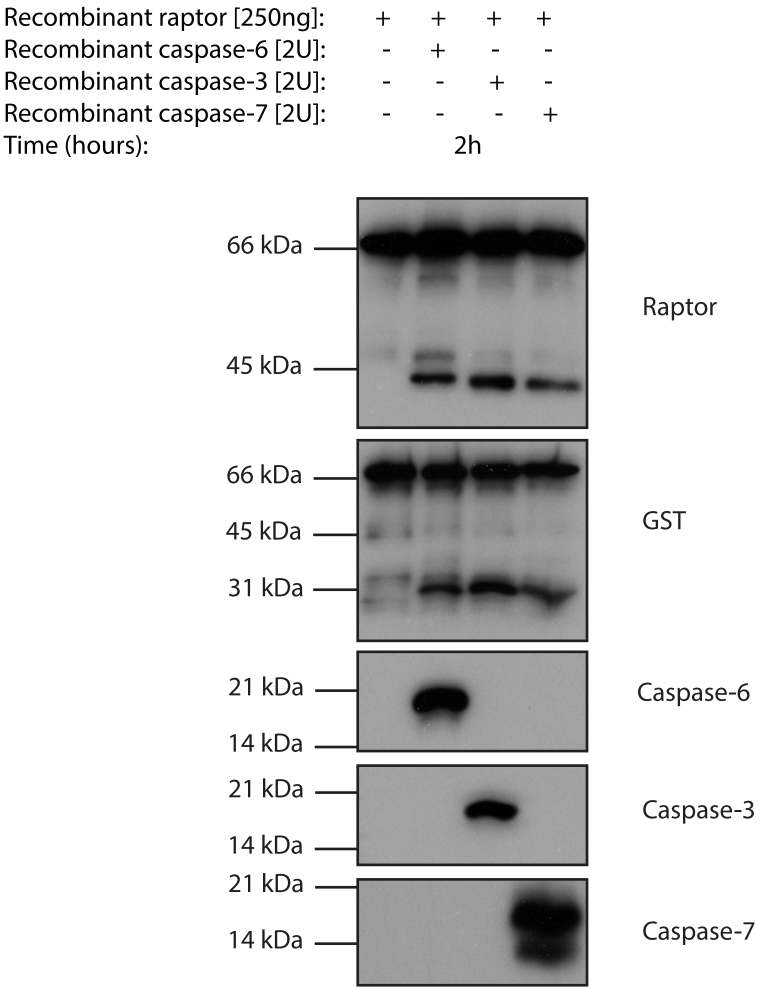

Recombinant raptor [250ng]: + + + +
Recombinant caspase-6 [2U]: - + - -
Recombinant caspase-3 [2U]: - - + -
Recombinant caspase-7 [2U]: - - - +
Time (hours): 2h
66 kDa
45 kDa
Raptor
66 kDa
45 kDa
31 kDa
GST
21 kDa
14 kDa
Caspase-6
21 kDa
14 kDa
Caspase-3
21 kDa
14 kDa
Caspase-7
