# Supplementary figures and images for: Caspase-mediated cleavage of raptor participates in the inactivation of mTORC1 during cell death
Source: Cell Death Discov. 2016 Apr 18;2:16024–. doi: 10.1038/cddiscovery.2016.24 (PMC4979510; doi:10.1038/cddiscovery.2016.24)

Figure S4

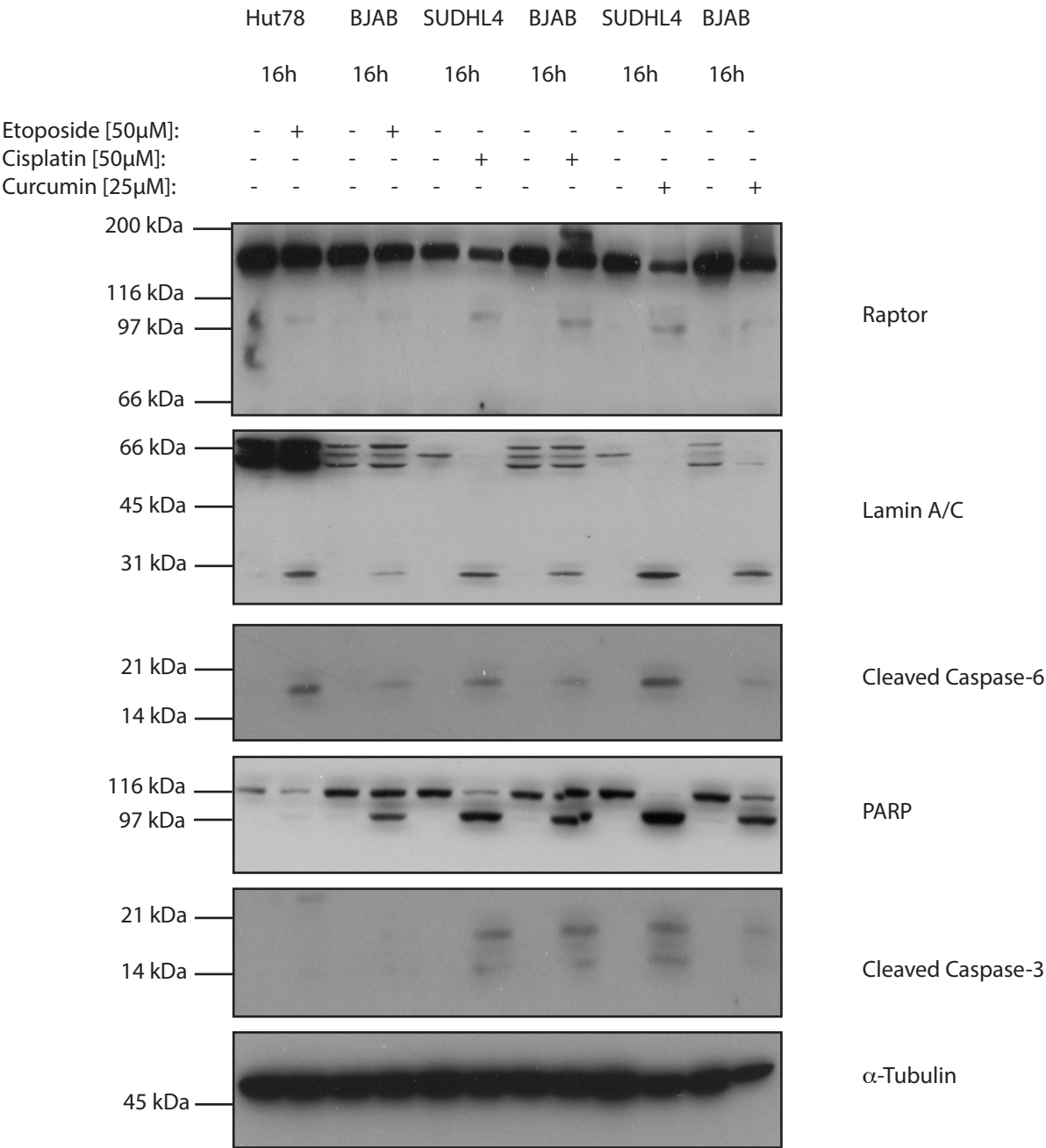

Supplement: Supplementary Figure 4 [file cddiscovery201624-s4.pdf]

Figure S5

A

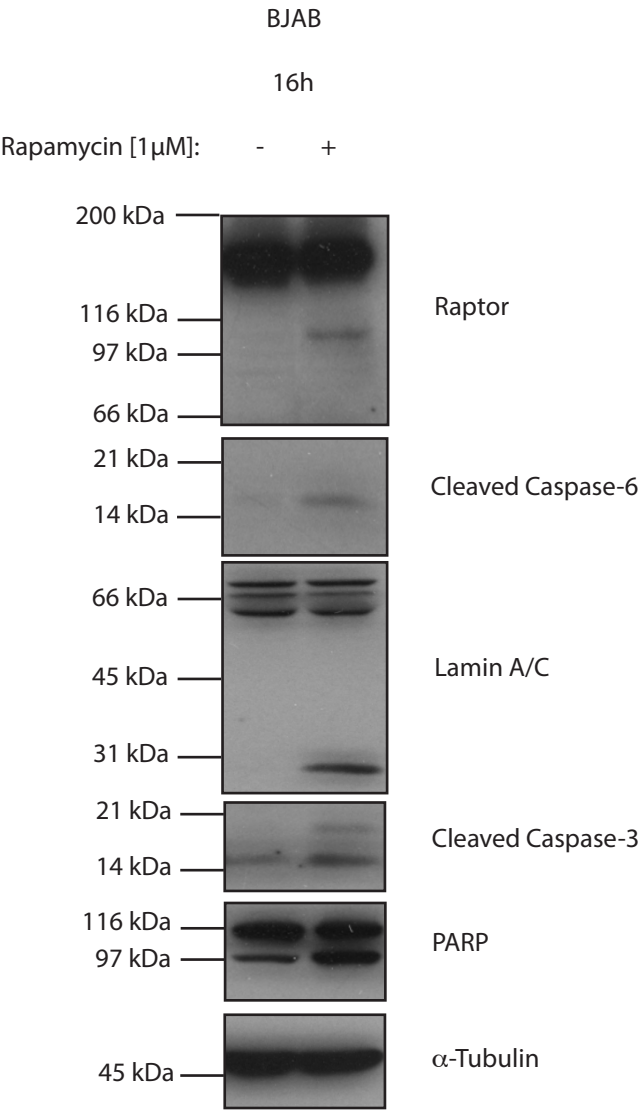

B

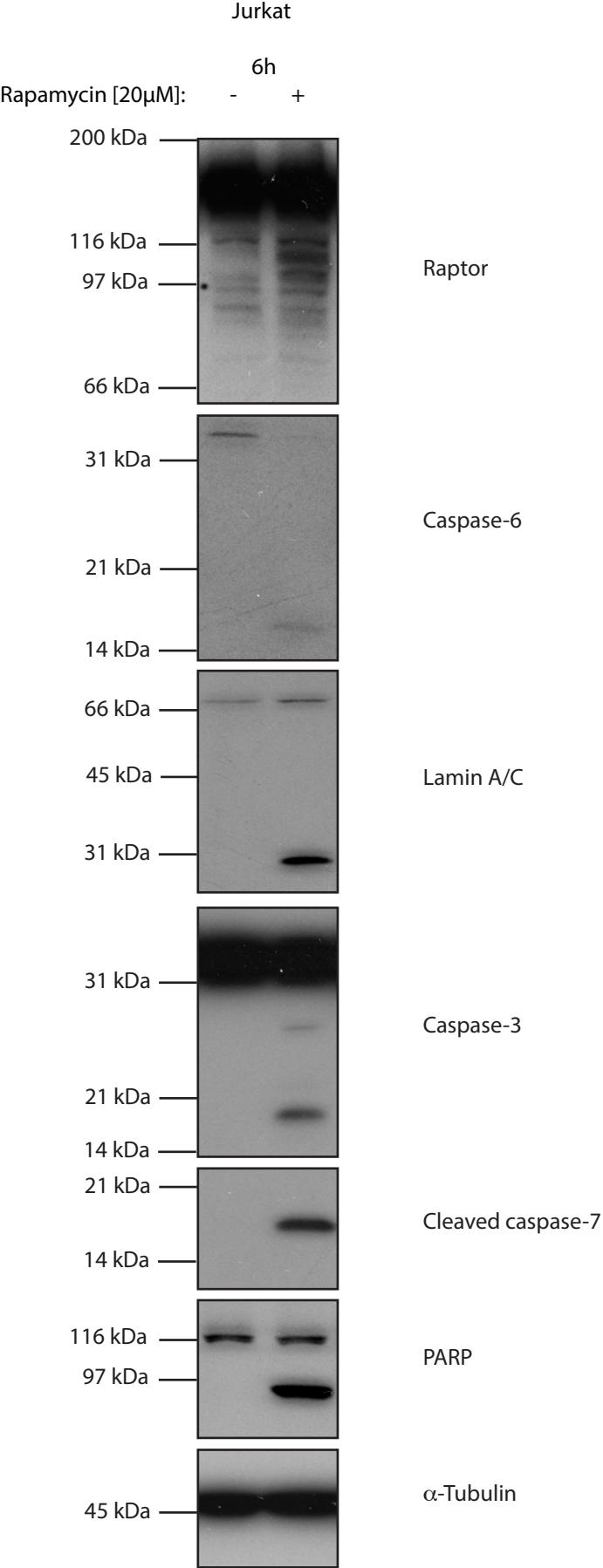

C

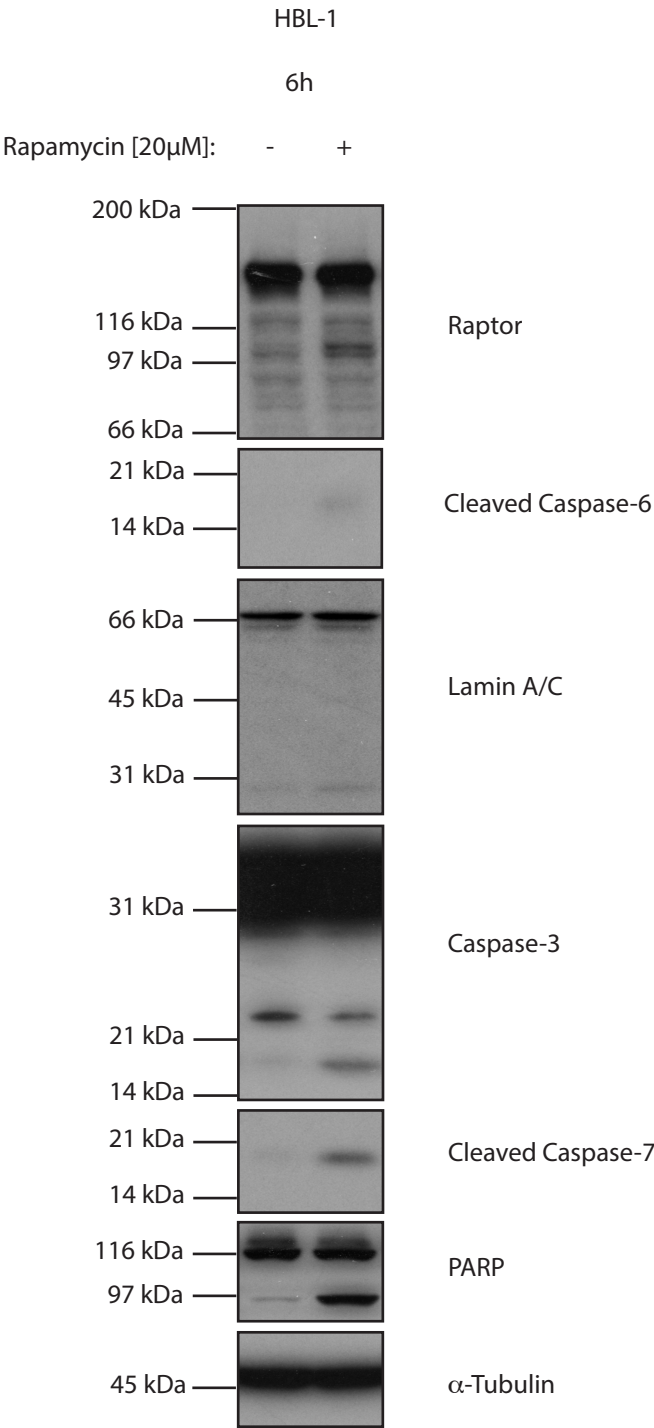

Supplement: Supplementary Figure 5 [file cddiscovery201624-s5.pdf]
