# Supplementary material for: Caspase-mediated cleavage of raptor participates in the inactivation of mTORC1 during cell death
Source: Cell Death Discov. 2016 Apr 18;2:16024–. doi: 10.1038/cddiscovery.2016.24 (PMC4979510; doi:10.1038/cddiscovery.2016.24)
Supplement: Supplementary Information [file cddiscovery201624-s6.doc]

**Supplementary figure legends**

**Figure S1** Raptor is not cleaved in BMDMø after caspase-1 activation.

Caspase-1 activation in BMDMø by nigericin or ATP after LPS priming. Cleaved caspase-1 (secreted) was detected in the supernatants, whereas raptor, Pro-IL-1β and α-tubulin were detected in the cell lysates.

**Figure S2** Identification by mass spectrometry of a raptor N-terminal cleavage site mediated by caspase-6 *in vitro.*

**A**) Sequence coverage of tryptic peptides on recombinant raptor incubated with active recombinant caspase-6. **B**) Semitryptic peptides identified and their MASCOT scores and PSM (peptide spectrum matches) numbers. PSMs are a rough measure of abundance. **C**) Tandem MS spectrum of peptide LTDWNLPLAFMK generated by cleavage after the DEAD sequence.

**Figure S3** Recombinant caspase-3 and caspase-7 can also cleave the N-terminal recombinant raptor in a similar fashion than recombinant caspase-6.

The N-terminal recombinant raptor was incubated alone or with recombinant caspase-6, -3 or -7 for 2h at 37°C. Recombinant N-terminal raptor processing was analyzed on 8% or 12% SDS-PAGE.

**Figure S4** Etoposide, cisplatin and curcumin induce activation of caspases, which correlate with raptor cleavage.

Hut78, BJAB and SUDHL4 were treated with 50µM etoposide, 50µM cisplatin or 25µM curcumin for 16h. Cell lysates were analyzed on 8% or 12% SDS-PAGE.

**Figure S5** Rapamycin (1µM or 20µM) induces raptor cleavage, which correlates with activation of caspases.

**A**) BJAB cells were incubated with 1µM of rapamycin for 16h. **B**) Jurkat T cells were incubated with 20µM of rapamycin for 6h. **C**) HBL-1 B cells were incubated with 20µM of rapamycin for 6h. Cells lysates were submitted to 8% or 12% SDS-PAGE for protein analysis.
